# NR4A1 promotes LEF1 expression in the pathogenesis of papillary thyroid cancer

**DOI:** 10.1038/s41420-022-00843-7

**Published:** 2022-02-02

**Authors:** Cen Jiang, Jianli He, Sunwang Xu, Qi Wang, Jinke Cheng

**Affiliations:** 1 State Key Laboratory of Oncogenes and Related Genes, Renji Hospital Affiliated; Shanghai Key Laboratory for Tumor Microenvironment and Inflammation, Department of Biochemistry and Molecular Cell Biology, Shanghai Jiao Tong University School of Medicine, 200025 Shanghai, China; 2 Department of Thyroid and Breast Surgery, The First Affiliated Hospital of Fujian Medical University, 350005 Fuzhou, China; 3 Department of Urology, Renji Hospital, Shanghai Jiao Tong University School of Medicine, 200127 Shanghai, China

**Keywords:** Thyroid cancer, Cell growth

## Abstract

The morbidity of papillary thyroid cancer (PTC) is on the rise, but its pathogenesis is still poorly understood. NR4A1 is a transcription factor primarily involving a wide range of pathophysiological responses, but its relationship with PTC malignancy remains unclear. This study demonstrates that high NR4A1 expression is strongly associated with poor survival outcomes in PTC patients. The depletion of NR4A1 significantly inhibited the proliferation of PTC cells by negating the LEF1-mediated oncogenic alteration. Mechanistically, NR4A1 directly binds to the promoter region of LEF1 and leads to crosstalk with histone acetylation and DNA demethylation to transcriptionally upregulate LEF1 expression, subsequently promoting downstream growth-related genes expressions in PTC. In the light of our findings, NR4A1 may be an emerging driving factor in PTC pathogenesis and progression.

## Introduction

In the last decades, the incidence of thyroid cancer has continuously and sharply increased. Papillary thyroid cancer (PTC) is the most common type of thyroid cancer, accounting for more than 80% of all thyroid cancers [1, 2]. PTC exhibits a gentle tumor biological behavior and an excellent survival outcome, but surprisingly, the morbidity of PTC is still on the rise in recent years [3, 4]. The pathogenesis of the origin and progression of PTC is still poorly understood. Several studies have investigated the genetic alteration of PTC, including the BRAF V600E mutation and RET/PTC rearrangements [5, 6]. Despite these advances, the underlying molecular mechanism of the pathogenesis of PTC needs to be further explored.

NR4A1 (also named NUR77, NGFIB, or TR3) is a transcription factor of the steroid/thyroid hormone receptor superfamily that controls the expression of downstream targets in a ligand-independent manner [7], regulating apoptosis, proliferation, cell cycle, endocrine system, inflammation, cell metabolism, and other physiological processes in normal or malignant cells [8, 9]. The function of NR4A1 in tumorigenesis is controversial. Previous studies have shown that NR4A1 acts as a tumor suppressor in both lymphoid and myeloid cells, resulting in growth inhibition and induction of apoptosis [10, 11]. In contrast, NR4A1 promotes proliferation and prevents apoptosis in colon and pancreatic cancers [12, 13]. Furthermore, the elevated expression of NR4A1 is also a prognostic marker for predicting adverse clinical outcomes in non-small-cell lung carcinoma and breast cancer [14, 15]. These findings reveal the complicated role NR4A1 plays in cancer development. Although a previous study has reported that lithium treatment-induced upregulation of NR4A1 in follicular thyroid carcinomas inhibited cell growth and triggered apoptosis [16], the contribution of NR4A1 to PTC pathogenesis has yet to be elucidated.

In the present study, the oncogenetic role of NR4A1 in PTC was investigated. The results showed that NR4A1 expression was upregulated in PTC tissues and was significantly associated with poor survival outcomes of PTC patients. Mechanistically, NR4A1 deletion in PTC cells could inhibit cell proliferation by negating the transcription of the oncogenic protein lymphoid enhancer factor-1 (LEF1) in a transcription factor binding motif dependent and epigenetic modification sensitive approach. Our work revealed NR4A1 as an emerging driving factor in PTC pathogenesis.

## Results

### Higher NR4A1 expression is associated with poor outcomes in PTC patients

**Fig. 1:**
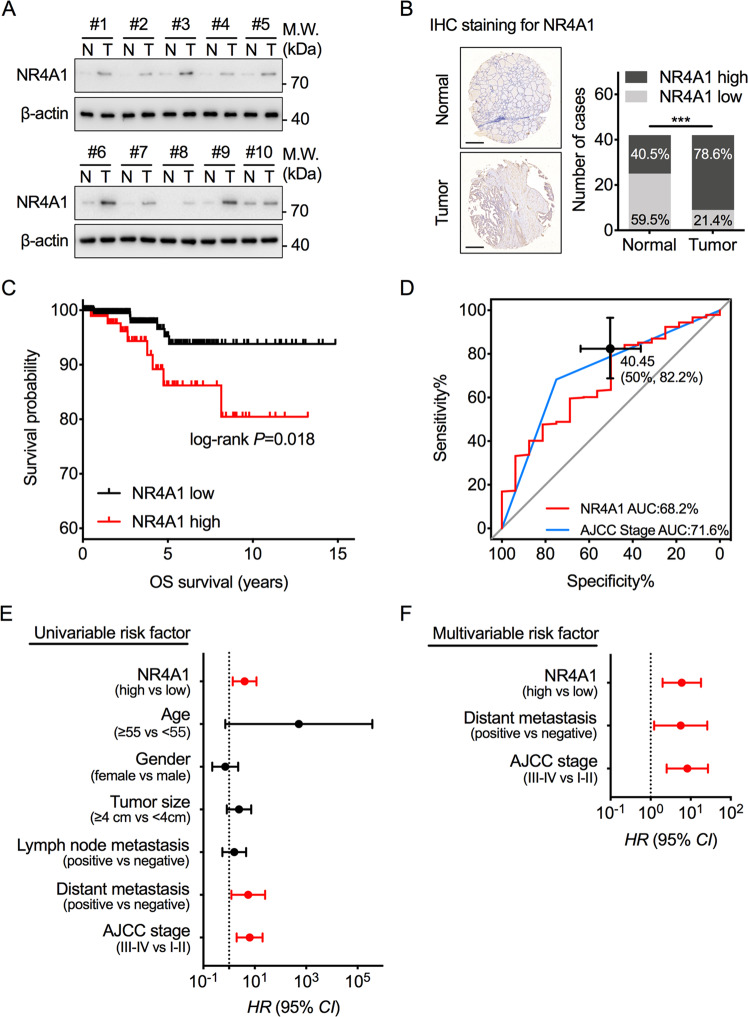
NR4A1 is upregulated in PTC and is associated with poor outcomes. **A** Western blot analysis of NR4A1 protein levels in ten representative paired PTC tumorous (T) tissues and adjacent normal tissues (N). **B** Representative images and semiquantitative analyses of immunohistochemistry (IHC) staining for NR4A1 in a tissue microarray (TMA) with 42 cases of paired PTC tumorous and non- tumorous tissues. Chi-square test, ***P < 0.001. Scale bars = 500 μm. **C** Overall survival (OS) was compared between PTC patients with low and high expression of NR4A1 in TCGA cohort, Log-rank test. **D** Receiver operating characteristic (ROC) analysis was conducted based on the expression of NR4A1 and AJCC stage for survival outcomes in the TCGA cohort. **E** Univariate Cox regression analysis was performed in the TCGA cohort. **F** Multivariate Cox regression analysis was performed in the TCGA cohort. The bars in (**E**) and (**F**) correspond to 95% confidence intervals (CI). The red represents statistical significance in Cox regression analysis.

To examine the NR4A1 expression status in PTC tissues, we compared the NR4A1 protein levels in PTC fresh tumorous tissues and adjacent normal tissues. We found that NR4A1 is upregulated in PTC tissues (Fig. 1A). To confirm this result, NR4A1 expression was analyzed in a PTC tissue microarray (TMA) containing 42 paired tumorous and adjacent normal thyroid tissues. NR4A1 was upregulated in 78.6% samples of PTC tumorous tissues, but only in 40.5% samples of adjacent tissues (Fig. 1B). These data indicated that NR4A1 is associated with tumorigenesis in PTC.

Furthermore, the relationship between NR4A1 expression and survival outcomes in PTC patients was evaluated. The NR4A1 transcript data and matched clinicopathological features of PTC samples obtained from The Cancer Genome Atlas (TCGA) demonstrated higher expression of NR4A1 was strongly associated with shorter overall survival (OS) in PTC patients (Fig. 1C). Receiver operating characteristic (ROC) curve analysis was utilized to compare the expression of NR4A1 and AJCC stage in predicting the survival outcomes of PTC patients. The results suggest that the area under curve (AUC) of NR4A1-based prediction was similar to the AJCC stage-based model (Fig. 1D). The optimal cut-off point for NR4A1 predicting poor outcomes was 40.45 (FPKM value), determined by the Youden Index method (Fig. 1D). In addition, univariate (Fig. 1E) and multivariate (Fig. 1F) regression analyses demonstrated that NR4A1 expression was an independent predictor of survival outcomes of PTC patients with significant hazard ratios. Conjointly, the study results indicate that the upregulated NR4A1 plays an oncogenic role in PTC and is clinically associated with poor survival outcomes.

### NR4A1 promotes tumor cell proliferation in PTC

**Fig. 2:**
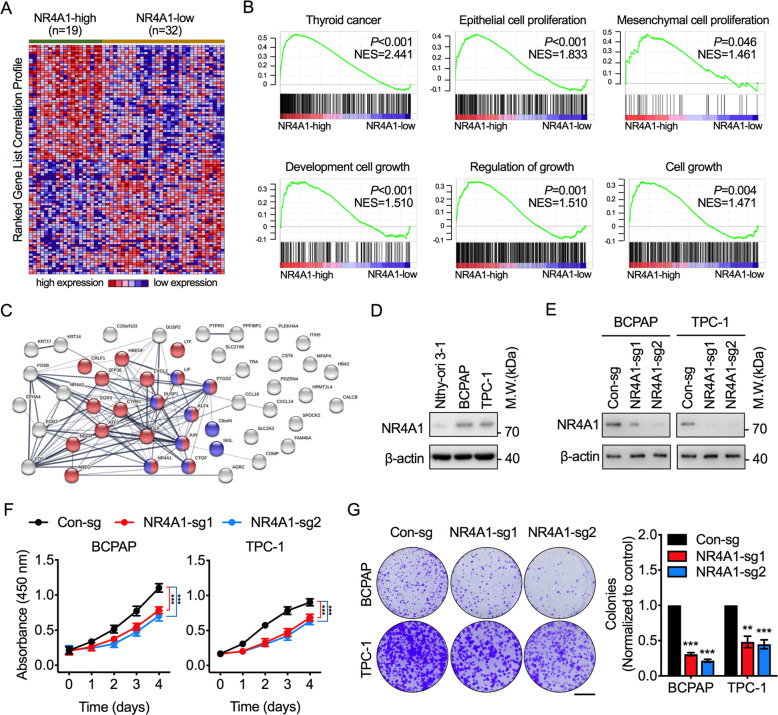
NR4A1 promotes PTC tumor cell proliferation. **A** Gene Set Enrichment Analysis (GSEA) was utilized to identify the differentially enriched signatures between high and low NR4A1 expression in the GSE27155 transcriptome dataset. The heatmap showed the top 50 top enriched and depleted genes in high NR4A1 expression group. **B** GSEA showed cell proliferation and growth-related biological functions enriched in response to higher NR4A1 expression. NES, Normalized Enrichment Score. **C** STRING analysis was performed to cluster the top enriched genes in the high NR4A1 expression group by similar biological functions. Blue and red nodes indicated clustering based on cell cycle and cell proliferation features. **D** Western blot analysis to compare NR4A1 expression in normal thyroid cells (Nthy-ori 3-1) and PTC cells (BCPAP and TPC-1). **E** Western blot analysis to validate NR4A1 knockout efficiency of NR4A1 in BCPAP and TPC-1 cells. **F** Cell viability assay of BCPAP and TPC-1 cells with or without NR4A1 knockout. One-way ANOVA test, ***P < 0.001. **G** Representative and quantification results of the colony formation assays for BCPAP and TPC-1 cells with or without NR4A1 knockout. Scale bar = 10 mm. Paired Student’s *t* test, **P < 0.01, ***P < 0.001.

To explore the potential effect of NR4A1 on the biological phenotype of PTC, the Gene Set Enrichment Analysis (GSEA) was performed, and the gene signatures of PTC samples with high and low NR4A1 expression were compared to identify the biological differences in the GEO dataset of GSE27155 (Fig. 2A). The GSEA results demonstrate that PTC samples with higher NR4A1 expression were significantly enriched in gene signatures related to cell proliferation and cell growth (Fig. 2B). The top-ranked genes from the GSEA were found to be clustered in the network STRING analysis and enriched in cell proliferation and cell cycle-associated pathways in PTC tissues with higher NR4A1 expression (Fig. 2C). These results suggest that NR4A1 might be essential for PTC proliferation and growth.

Two PTC cell lines, BCPAP and TPC-1 cells, featuring high NR4A1 expression compared to normal thyroid cell line Nthy-ori 3-1 (Fig. 2D), were used to further investigate the biological function of NR4A1 on PTC. NR4A1 knockout was induced in BCPAP and TPC-1 cells by the CRISPR/Cas9 approach (Fig. 2E). Cell viability assays demonstrated that knockout of NR4A1 significantly inhibits cell proliferation in PTC cells (Fig. 2F). In accordance with the above findings, colony formation assays confirmed that NR4A1 knockout lowered the colonization ability of PTC cells (Fig. 2G). Conjointly, these results demonstrate that NR4A1 promotes PTC cell proliferation and tumor aggressiveness.

### NR4A1 is required for the expression of LEF1 in PTC

**Fig. 3:**
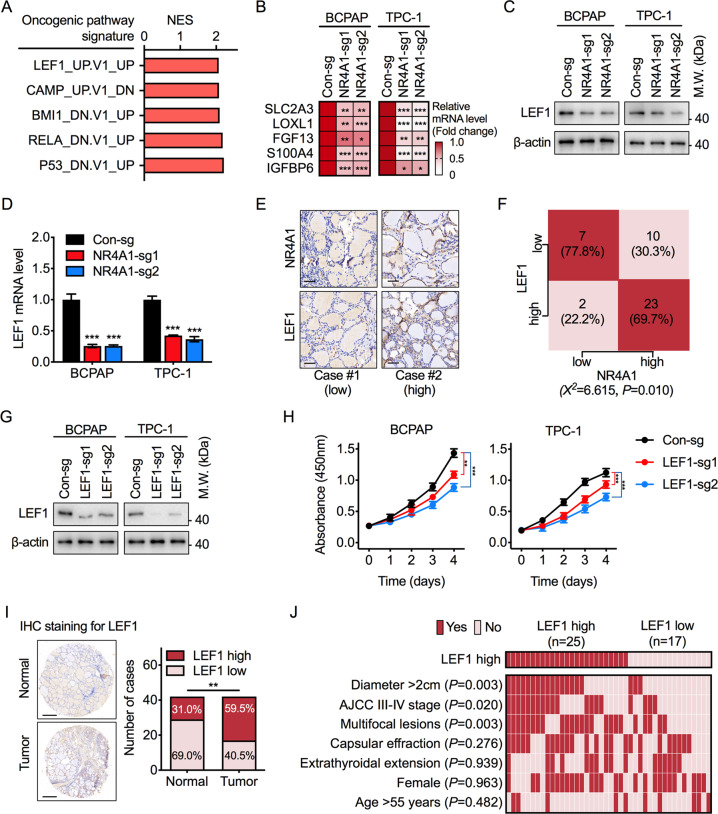
NR4A1 is associated with LEF1 expression in PTC. **A** GSEA analysis of GSE27155 by Oncogenic Pathway database to identify the potentially enriched signatures in higher NR4A1 expression of PTC tissues. **B** The heatmap showed the RT-qPCR results of LEF1 target gene expression in NR4A1 knockout and control BCPAP and TPC-1 cells. Unpaired Student’s *t* test, *P < 0.05, **P < 0.01, ***P < 0.001. **C** Western blot analysis of LEF1 protein levels in NR4A1 knockout and control cells. **D** RT-qPCR results of LEF1 mRNA levels in NR4A1 knockout and control cells. The NR4A1 knockout group was compared to the control group, Student’s *t* test, ***P < 0.001. **E** Representative IHC stainings of NR4A1 (upper) and LEF1 (lower) proteins in PTC tumorous tissues with high or low NR4A1 expression. Scale Bars = 50 μm. **F** Statistical analysis was conducted based on the NR4A1, and LEF1 protein expression in the TMA with 42 cases of PTC. Chi-square test. **G** Western blot analysis to validate LEF1 knockout efficiency in BCPAP and TPC-1 cells. **H** Cell viability assay via CCK8 method to compare the proliferation rate between LEF1 depleted and control BCPAP and TPC-1 cells. One-way ANOVA test, **P < 0.01, ***P < 0.001. **I** Representative images and semiquantitative analyses of IHC staining of LEF1 protein in a TMA of paired PTC tumorous tissues and adjacent normal tissues. Chi-square test, **P < 0.01. Scale bars = 500 μm. **J** The heatmap illustrated the association of different clinical characters in PTC patients with high or low LEF1 expression. Statistical significance was assessed by the Chi-square test.

The GSEA results of the GSE27155 dataset provided insight into the potential enrichment of oncogenic pathways. A significant and specific LEF1-dependent signature was enriched in the high NR4A1 expression group (Fig. 3A). In contrast, the transcription of LEF1 targeted genes was strongly diminished in NR4A1 knockout cells (Fig. 3B). Given the importance of LEF1 on driving tumor initiation and progression [17–19], we surmised that NR4A1 might stimulate PTC tumor aggressiveness through the LEF1 pathway.

Hence, the effect of NR4A1 on LEF1 expression in PTC cells was investigated. The results showed that NR4A1 knockout could decrease LEF1 expression in both proteins (Fig. 3C) and mRNA levels (Fig. 3D). Remarkably, LEF1 expression in PTC tumorous tissues was strongly positively correlated with NR4A1 proteins (Fig. 3E, F). Further confirmed by cell viability assays, LEF1 depletion indeed reduced the proliferation rate of PTC cells (Fig. 3G, H), producing similar results to NR4A1 knockout.

Moreover, the expression of LEF1 in PTC tissues was assessed by the immunohistochemistry (IHC) staining of LEF1 in the TMA of paired PTC tumorous and adjacent tissues. The results confirmed that LEF1 is highly expressed in PTC tissues (Fig. 3I). Furthermore, the relationship between the expression of LEF1 and different clinicopathological features of PTC was evaluated. LEF1 expression was positively correlated with tumor size, advanced tumor stage, and the number of tumorous lesions (Fig. 3J). Taken together, these findings demonstrate that NR4A1 is associated with LEF1 expression in PTC cells, and LEF1 promotes PTC cell proliferation.

### NR4A1 crosstalk with epigenetic modifications to activate LEF1 transcription

**Fig. 4:**
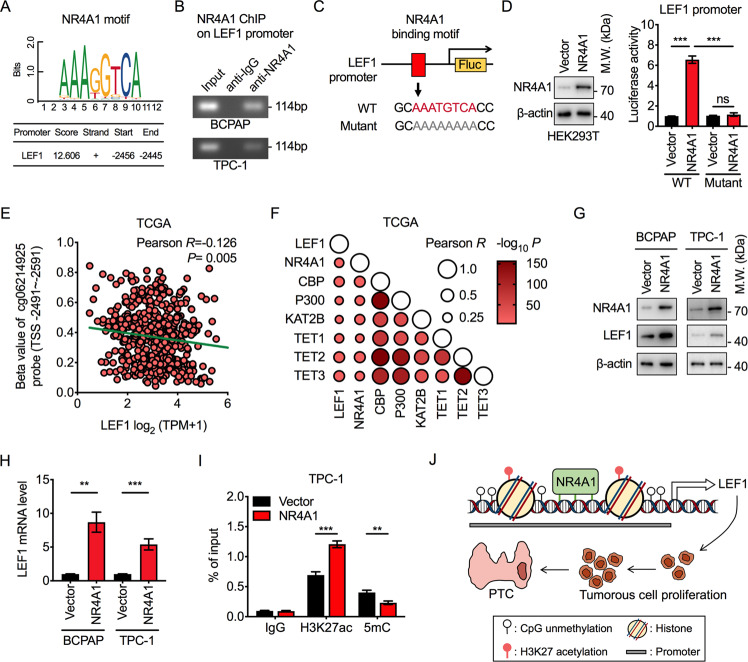
NR4A1 crosstalk with epigenetic changes to transcriptionally increase LEF1. **A** Schematic diagram of the NR4A1-binding motif in the promoter of LEF1, predicted by the JASPAR database. **B** ChIP-PCR results of NR4A1 enrichment at the promoter of LEF1 in BCPAP and TPC-1 cells. **C** Schematic diagram of the luciferase reporter constructs contains the promoter of LEF1 with conserved wild-type (WT) NR4A1-binding motif and mutant strategy. **D** HEK293T cells were transfected with empty vector or NR4A1-overexpressed plasmids (left panel) to analyze the luciferase reporter activity driven by WT or mutant LEF1 promoter (right panel). Student’s *t* test, ***P < 0.001, ns, not significant. **E** Correlation between the methylation level of the probe cg06214925 (located near NR4A1-binding motif within LEF1 promoter) and the transcription level of LEF1 in PTC tumorous tissues in TCGA database. The linear correlation was determined by Pearson Correlation Coefficient. **F** Correlation between the expression levels of LEF1, NR4A1, histone acetyltransferase, and DNA demethylase in PTC tumorous tissues in TCGA database, performed by Pearson Correlation Coefficient analysis. **G** Western blot analysis of NR4A1 and LEF1 protein levels in BCPAP and TPC-1 cells transfected with empty vector or NR4A1-overexpressed plasmids. **H** RT-qPCR results of LEF1 mRNA levels in empty vector or NR4A1-overexpressed cells. Student’s *t* test, **P < 0.01, ***P < 0.001. **I** ChIP-qPCR analysis of H3K27ac and 5mC occupancy at the LEF1 promoter region around the NR4A1-binding site in TPC-1 cells. Student’s *t* test, **P < 0.01, ***P < 0.001. **J** Model depicting the role of NR4A1 in promoting the PTC cell proliferation via transcriptionally controlling LEF1.

Next, the relationship between NR4A1 and LEF1 transcription was investigated. A conserved NR4A1-binding motif (AAATGTCA) was identified at the promoter region of LEF1, as predicted by the JASPAR database (Fig. 4A). ChIP-PCR analysis in PTC cells confirmed that NR4A1 could bind to the promoter of LEF1 (Fig. 4B). In addition, we generated the luciferase reporter construct containing wild-type (WT) NR4A1-binding site or the mutant region (TGTC to AAAA) (Fig. 4C) and performed luciferase assay reporter assay to confirm the direct regulation of NR4A1 on LEF1 transcription. The results revealed that the wild-type LEF1 promoter induced substantial luciferase activity while the mutant region completely inhibited the activity (Fig. 4D). The above data suggest that NR4A1 directly binds to the promoter region of LEF1 and transcriptionally upregulates its expression.

Epigenetic modifications often collaborate transcription factors in driving target genes’ transcription activation, such as DNA demethylation and histone acetylation [20]. The DNA methylation levels near the NR4A1-binding site of the LEF1 promoter were negatively correlated with LEF1 transcription levels (Fig. 4E). In contrast, the expression of LEF1 was positively correlated with the expression of NR4A1, histone acetyltransferase, and DNA demethylase in the TCGA database (Fig. 4F). Subsequently, the epigenetic modifications in NR4A1-driven transcription activation of LEF1 were further analyzed. Ectopic expression of NR4A1 in PTC cells could significantly increase LEF1 expression of mRNA and protein levels (Fig. 4G, H). More importantly, NR4A1-activated LEF1 expression was accompanied by increased H3K27 acetylation (H3K27ac) and reduced CpG methylation (5mC) around the NR4A1-binding site in the LEF1 promoter, representing for the transcription activation of LEF1 (Fig. 4I). The above results confirm the NR4A1 crosstalk with histone modifications and DNA methylation to activate LEF1 expression in PTC.

## Discussion

This study aimed to advance our understanding of the role of NR4A1 in PTC tumorigenesis. We found that NR4A1 was upregulated in PTC tissues and was strongly associated with poor overall survival in PTC patients. Furthermore, NR4A1 could promote PTC proliferation via controlling the transcription of oncogenic protein LEF1 in an epigenetic-dependent approach (Fig. 4J). Our findings uncover an oncogenic function for NR4A1 in the papillary histotype of thyroid cancer.

Several studies have shown that the etiology of PTC is controlled by multiple transcription factors regulating distinct developmental aspects [21, 22]. This study demonstrates that NR4A1 plays a crucial role in the development of PTC by regulating cell proliferation. NR4A1 was initially known as an immediate-early gene induced in response to growth factors, calcium, inflammatory cytokines, peptide hormones, phorbol esters, or other stimuli [23, 24]. Recently, it was reported that NR4A1 mRNA levels could be regulated by the thyroid hormone in the pituitary [25], indicating that NR4A1 expression might play an essential role in the thyroid physiological process and NR4A1 may regulate cellular susceptibility to tumorigenesis.

Multiple studies have described the correlation between cell proliferation and NR4A1. Notably, NR4A1 is necessary and sufficient for NK6 homeobox 1-mediated β-cell proliferation. Furthermore, global NR4A1 knockout leads to a decrease in β-cell area in neonatal and young mice [26]. Consistently, NR4A1 expression can mediate bile acid-induced cell growth in human colon and liver cancer cells by modulating the expression of survival genes [27]. NR4A1 regulates cell proliferation in a cell type and tissue-dependent manner. In the present study, the oncogenic role of NR4A1 in driving PTC tumor cell proliferation and clinical progression of PTC patients is described.

Moreover, recent studies revealed that the activation of LEF1 expression in various tumors promotes the transcription of genes that are necessary for growth [28–30]. A direct connection between NR4A1 and LEF1 in PTC was found in the present study, where LEF1-induced gene signatures were strongly activated in PTC tissues with high NR4A1 expression. The activity of NR4A1 is mostly due to direct activation or repression of multiple target genes. NR4A1 can be observed as organized homodimers or even monomers directly binding to the promoter of target genes containing the NBRE motif and driving or preventing transcription initiation [31]. This study further confirms that NR4A1 directly binds to the LEF1 promoter region and induces crosstalk with increased H3K27ac and decreased CpG methylation to collaboratively activate LEF1 transcription. However, whether the regulation of LEF1 by NR4A1 is common in other types of mammalian cells and tissues remains unknown and requires further research.

In conclusion, this study confirms that NR4A1 plays an oncogenic role in promoting PTC progression and mediating poor survival outcomes of PTC patients via crosstalking with epigenetic modifications to activate LEF1 expression and its oncogenic biofunctions. In light of our findings, targeting NR4A1-mediated LEF1 expression might be a potential therapeutic target to prevent PTC initiation and progression.

## Materials and methods

### Cell lines

Human PTC cell lines BCPAP and TPC-1 and normal thyroid cell line Nthy-ori 3-1 were obtained from the Cell Bank of Type Culture Collection of Chinese Academy of Science, and the human embryonic kidney 293 T (HEK293T) cell was obtained from the American Type Culture Collection. TPC-1, BCPAP, Nthy-ori 3-1 and HEK293T were cultured in DMEM medium (Hyclone, USA), RPMI-1640 medium (Hyclone, USA), RPMI-1640 medium (Hyclone, USA), RPMI-1640 medium (Hyclone, USA), and DMEM medium (Hyclone, USA), respectively, supplemented with 10% fetal bovine serum (Gibco, USA) and 1% penicillin-streptomycin (Gibco, USA) at 37°C in a humidified 5% CO_2_ (v/v) atmosphere. All cell lines have been tested for mycoplasma contamination.

### Lentiviral infection and knockout cell lines generation

NR4A1 and LEF1 knockout cell lines were generated by CRISPR/Cas9 method. For lentiviral infection, the virus released from HEK293T cells was collected and used to infect BCPAP and TPC-1 cells. The infected cells were selected with puromycin to generate knockout cell pools. Single-guide RNAs (sgRNAs) targeting NR4A1 and LEF1 were cloned into LentiCRISPR-V2 vector. The sgRNAs sequences as indicated: NR4A1-sg1 (TACACCCGTGACCTCAACCA), NR4A1-sg2 (GGCTAACAAGGACTGCCCTG), LEF1-sg1 (GTACCCGGAATAACTCGAGT), LEF1-sg2 (GTGTTACAATAGCTGGATGA).

### RNA extraction and real-time quantitative PCR (RT-qPCR)

Total RNA was extracted from cells using TRI Reagent (Sigma-Aldrich, USA), and 1 μg of total RNA was reverse transcribed using 1st cDNA Synthesis Kit (Yeasen, China) to detect relative mRNAs. RT-qPCR was performed in triplicated on an Applied Biosystems ViiA TM 7 Real-Time PCR system (Applied Biosystems, USA). The Ct values obtained from different samples were compared using the 2^−ΔΔCt^ method, and ACTB served as internal reference gene. The primers as indicated: LEF1, forward: 5′-GACGAGATGATCCCCTTCAA-3′, reverse: 5′-CGGGATGATTTCAGACTCGT-3′; SLC2A3, forward: 5′-GCTGGGCATCGTTGTTGGA-3′, reverse: 5′-GCACTTTGTAGGATAGCAGGAAG-3′; LOXL1, forward: 5′-GTGCAGCCTGGGAACTACAT-3′, reverse: 5′-CAGAAACGTAGCGACCTGTG-3′; FGF13, forward: 5′-ACAAGCCTGCAGCTCATTTT-3′, reverse: 5′-AGCACGCCAGAGACACTTCT-3′; S100A4, forward: 5′-GATGAGCAACTTGGACAGCA-3′, reverse: 5′-CTTCCTGGGCTGCTTATCTG-3′; IGFBP6, forward: 5′-GAATCCAGGCACCTCTACCA-3′, reverse: 5′-CTGCAGCACTGAGTCCAGAT-3′; ACTB, forward: 5′-CATGTACGTTGCTATCCAGGC-3′, reverse: 5′-CTCCTTAATGTCACGCACGAT-3′.

### Western blot

Cell lysates were harvested and quantified via BCA Protein Assay Kit (Thermo Fisher Scientific, USA). Secondary antibodies were labeled with HRP (Sigma-Aldrich, USA) and the signals were detected using ECL Kit (Millipore, USA). A β-Actin served as an internal control for the whole-cell lysates. Antibody against NR4A1 (ab109180, dilution 1:1000) and LEF1 (ab85052, dilution 1:1000) were purchased from Abcam (USA), β-actin (A1978, dilution 1:10000) were purchased from Sigma-Aldrich (USA).

### Luciferase assay

The promoter sequences (−3000 to −1 bp) of LEF1 genome were cloned to pGL3-basic promoter vector. Luciferase assays were performed in HEK293T cells with pGL3-LEF1 promoter wild-type luciferase reporter or NR4A1-binding motif mutant luciferase reporter. 500 ng reporter plasmid were transfected together with 50 ng pRL-TK-Renilla-luciferase plasmid to normalize for transfection efficiency. For luciferase assays in NR4A1 overexpression plasmid-transfected cells, cells were transfected with the 1 μg NR4A1 overexpression plasmids or 1 μg empty vector plasmids together with reporter plasmid for 24 h, and then the Fluc and Rluc activities were determined according to the manufacturer’s protocol of Dual-Luciferase Reporter Assay System (Promega, USA). The reporter activity was calculated by the ratio of Fluc to Rluc, and then normalized to the control group.

### Cell viability assay

Cell viability was assessed by Cell Counting Kit-8 (Dojindo, Japan) assay. Cells were seeded at 4000 cells/well in 96-well plates with 100 μl culture medium. 10 μl of CCK-8 solution was added to the cells at the indicated time points and cells were incubated for 1 h at 37°C. After that, the reaction product was quantified by the Synergy 2 microplate reader (Biotek, USA) at the absorbance of 450 nm.

### Colony formation assay

For colony formation, cells were seeded at 200 cells/well in 6-well plates with 2 ml culture medium per well for culturing until colonies were visible. Then, the colonies were fixed with 4% paraformaldehyde, stained with 0.1% crystal violet, and photographed by scanner.

### Chromatin immunoprecipitation (ChIP)

ChIP assay was performed using SimpleChIP Enzymatic Chromatin IP Kit (#9002, Cell Signaling Tech, USA) following the manufacturer’s protocol. Chromatin was immunoprecipitated with antibody of NR4A1 (NB100-56745, Novus, USA), H3K27ac (#8173, Cell Signaling Tech, USA), 5mC (SAB2702243, Sigma-Aldrich, USA) or IgG (#2729, Cell Signaling Tech, USA) overnight at 4°C. Antibody/chromatin complexes were recovered with Protein G agarose beads for 2 h, and finally eluted and purified. The purified DNA was performed with ChIP-PCR and run in Agarose gel, or ChIP-qPCR and quantify the relative occupancy. The primer for ChIP-PCR and ChIP-qPCR for LEF1 promoter as following: forward: 5′-TCCTCCTGCTGTTTCCAAAA-3′ reverse: 5′-CAGCACTTAGAAGGGGCTTT-3′.

### Gene set enrichment analysis (GSEA) and STRING analysis

The transcript profiles of PTC, obtained from Gene Expression Omnibus (GEO) under the series accession number of GSE27155, were used to conduct heatmap, GO analysis, and GSEA to identify gene signatures between groups with high and low NR4A1 expression. GSEA results are shown using normalized enrichment scores (NES), accounting for the size and degree to which a gene set in overrepresented at the top or bottom of the ranked list of genes (NES > 1, *P* < 0.05, and FDR < 0.25). A STRING network (https://string-db.org/) were used for visualization of the GSEA results with the top 50 positive ranked genes between high and low NR4A1 groups.

### Clinical samples

The ten paired PTC fresh tissues and a tissue microarray contain 42 paired formalin-fixed paraffin-embedded PTC tissues were collected from the Department of Thyroid and Breast Surgery, The First Affiliated Hospital of Fujian Medical University. The Ethics Committees in The First Affiliated Hospital of Fujian Medical University approved the study protocols. Written informed consents were obtained from all participants in this study. All the research was carried out in accordance with the provisions of the Helsinki Declaration of 1975.

### Histological analysis

For NR4A1 and LEF1 expression analysis, TMA slide was immunostained with anti-NR4A1 (NB100-56745, dilution 1:50, Novus, USA) or anti-LEF1 (HPA002087, dilution 1:100, Sigma-Aldrich, USA) overnight at 4°C and counterstained with hematoxylin. For analysis, the slides were scanned on Pannoramic DESK (3D HISTECH, Hungary). The protein expression was determined via the positive staining intensity evaluated by two independent pathologists. Negative or weak staining defined as low expression, intermediate or strong defined as high expression.

### Statistical analysis

Data from at least three independent experiments performed in triplicates are presented as the means ± SD. Error bars in the histograms represent SD. For statistical evaluation, paired or unpaired Student’s *t* test was applied to calculate the statistical probability between two groups, and one-way ANOVA analysis was used to calculate the statistical probability between three or more than three groups in this study. Chi-square test was used to analyze the correlation of qualitative data. For survival analysis, the Kaplan–Meier method and log-rank tests were applied to determine the overall survival. Statistical calculation was performed using SPSS 23.0, and a *P* value <0.05 was considered to be statistically significant.

## Data Availability

The data used to support the findings of this study are available from the corresponding author upon request.
